# Clinical factors associated with persistently poor diabetes control in the Veterans Health Administration: A nationwide cohort study

**DOI:** 10.1371/journal.pone.0214679

**Published:** 2019-03-29

**Authors:** Anastasia-Stefania Alexopoulos, George L. Jackson, David Edelman, Valerie A. Smith, Theodore S. Z. Berkowitz, Sandra L. Woolson, Hayden B. Bosworth, Matthew J. Crowley

**Affiliations:** 1 Center of Innovation to Accelerate Discovery and Practice Transformation (ADAPT), Durham Veterans Affairs Health Care System, Durham, NC, United States of America; 2 Division of Endocrinology, Duke University, Durham, NC, United States of America; 3 Department of Population Health Sciences, Duke University, Durham NC, United States of America; 4 Division of General Internal Medicine, Duke University, Durham NC, United States of America; 5 Department of Psychiatry & Behavioral Sciences, Duke University, Durham NC, United States of America; Weill Cornell Medical College Qatar, QATAR

## Abstract

**Objective:**

Patients with persistent poorly-controlled diabetes mellitus (PPDM) despite engagement in clinic-based care are at particularly high risk for diabetes complications and costs. Understanding this population’s demographics, comorbidities and care utilization could guide strategies to address PPDM. We characterized factors associated with PPDM in a large sample of Veterans with type 2 diabetes.

**Methods:**

We identified a cohort of Veterans with medically treated type 2 diabetes, who received Veterans Health Administration primary care during fiscal years 2012 and 2013. PPDM was defined by hemoglobin A1c levels uniformly >8.5% during fiscal year (FY) 2012, despite engagement with care during this period. We used FY 2012 demographic, comorbidity and medication data to describe PPDM in relation to better-controlled diabetes patients and created multivariable models to examine associations between clinical factors and PPDM. We also constructed multivariable models to explore the association between PPDM and FY 2013 care utilization.

**Results:**

In our cohort of diabetes patients (n = 435,820), 12% met criteria for PPDM. Patients with PPDM were younger than better-controlled patients, less often married, and more often Black/African-American and Hispanic or Latino/Latina. Of included comorbidities, only retinopathy (OR 1.68, 95% confidence interval (CI): 1.63,1.73) and nephropathy (OR 1.26, 95% CI: 1.19,1.34) demonstrated clinically significant associations with PPDM. Complex insulin regimens such as premixed (OR 10.80, 95% CI: 10.11,11.54) and prandial-containing regimens (OR 18.74, 95% CI: 17.73,19.81) were strongly associated with PPDM. Patients with PPDM had higher care utilization, particularly endocrinology care (RR 3.56, 95% CI: 3.47,3.66); although only 26.4% of patients saw endocrinology overall.

**Conclusion:**

PPDM is strongly associated with complex diabetes regimens, although heterogeneity in care utilization exists. While there is evidence of underutilization, inadequacy of available care may also contribute to PPDM. Our findings should inform tailored approaches to meet the needs of PPDM, who are among the highest-risk, highest-cost patients with diabetes.

## Introduction

The prevalence of diabetes in the U.S. is over 30 million [1]. Diabetes underlies 12% of deaths [2] and accounts for $327 billion in healthcare costs [3]. It is the main cause of microvascular complications (nephropathy, neuropathy and retinopathy); patients with diabetes also experience higher rates of macrovascular complications (heart disease and stroke) and a two-fold increase in mortality compared to other patients [4]. While the complications and costs of diabetes rise exponentially as hemoglobin A1c (HbA1c) increases [5–7], interruption of poor glycemic control reduces complications [8–10]. Although select individuals may benefit from looser HbA1c goals [11–13], maintenance of elevated HbA1c generally confers a heightened risk for complications that is modifiable with improved glycemic control.

Suboptimal access to quality healthcare underlies persistently poor diabetes control for some individuals. However, other people maintain an elevated HbA1c despite regularly receiving diabetes care, a scenario we have designated ‘persistent poorly-controlled diabetes mellitus’ (PPDM). We define PPDM as maintenance of HbA1c >8.5% for ≥1 year despite clinic-based diabetes care during this period. Given their persistently poor diabetes control, those with PPDM are likely among the highest-risk, highest-cost diabetes patients in any healthcare system.

Because diabetes control has a substantial impact on complications and costs in patients with high HbA1c [9], addressing PPDM should be a priority for healthcare providers and systems. However, the prevalence and clinical correlates of PPDM have only been characterized in small studies [14]. Likewise, we know little about current care utilization patterns in PPDM.

In this analysis, we sought to characterize the scope of PPDM among a national sample of Veterans with type 2 diabetes receiving primary care. We used nationwide Veterans Health Administration (VHA) data to examine demographics, comorbidities, medication use, care utilization, and disease control in PPDM, as compared to better-controlled diabetes patients.

## Methods

### Data source and sample

This cohort study used national VHA electronic health record (EHR) data, and was approved by the Institutional Review Board (IRB) of the Durham Veterans Affairs Health Care System (#01808). As this was a secondary analysis of existing data, a waiver of informed consent was obtained. Data were not fully anonymized before being accessed by our team.

The construction of the study cohort has been described previously [15–17]. Our sample comprised adults (age ≥18 years) with medically treated type 2 diabetes who received diabetes care within VHA during fiscal year (FY) 2012 (October 1, 2011-September 30, 2012) and FY 2013 (October 1, 2012-September 30, 2013). Diagnosis of medically-treated type 2 diabetes was established when patients had: 1) a pertinent International Classification of Diseases 9^th^ revision (ICD-9) code (250.x0 or 250.x2) associated with ≥1 inpatient visit and/or ≥2 outpatient visits during FY 2012; and 2) at least one filled outpatient prescription for an antihyperglycemic agent (VA drug classes HS501 or HS502) during FY 2012. Patients also required evidence of ongoing diabetes care during FY 2012, defined as ≥1 primary care clinic visit (determined using VHA stop codes 322, 323, 342, and 348) and ≥2 measured HbA1c values. Patients were assigned a “home” VHA facility based on their most frequently visited primary care clinic during FY 2012.

Patients were excluded if they: 1) were <18 years at the beginning of FY 2012; 2) had an ICD-9 code for type 1 diabetes (250.x1 or 250.x3); 3) had no visits with associated diabetes diagnoses during FY 2013 (as well as FY 2012); 4) had <2 available HbA1c measurements from FY 2012; 5) did not have a home VHA facility with at least 100 cohort members during FY 2012; 6) had a home VHA facility that was either >1000 miles from their home ZIP code or was not in one of the 50 US states/District of Columbia; or 7) had missing or biologically implausible (<13 or >85 kg/m^2^) body mass index (BMI) information.

### Diabetes control categories

**Fig 1:**
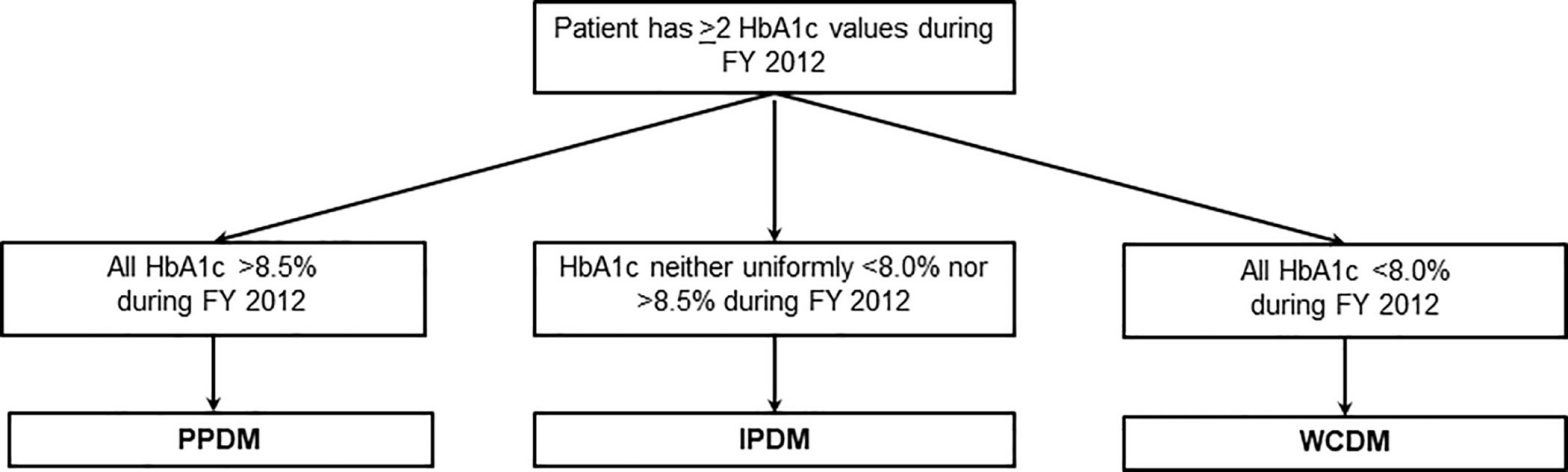
Algorithm for allocating patients into type 2 diabetes control categories. **Abbreviations:** FY = fiscal year; HbA1c = hemoglobin A1c; PPDM = persistent poorly-controlled diabetes mellitus; IPDM = intermittent poorly-controlled diabetes mellitus; WCDM = well-controlled diabetes mellitus.

To characterize factors associated with PPDM, we used all HbA1c measurements from FY 2012 to categorize patients as PPDM, ‘intermittent poorly-controlled diabetes mellitus’ (IPDM), or ‘well-controlled diabetes mellitus’ (WCDM). Our diabetes control subgroups patient allocation algorithm is in Fig 1.

We selected a conservative HbA1c upper limit of 8.0% to define WCDM because less stringent HbA1c goals may be warranted for patients with a history of hypoglycemia, multiple comorbidities, or advanced diabetes complications per American Diabetes Association (ADA) guidelines [12]. Patients whose FY 2012 HbA1c measurements were uniformly <8.0% were therefore categorized as WCDM. We chose 8.5% as our lower HbA1c cutoff for PPDM because an HbA1c >8.5% reflects poor control for most patients in the VHA system and beyond, even for individuals with lower life expectancy and significant microvascular complications [12, 18]. Patients whose FY 2012 HbA1c values were uniformly >8.5% were therefore categorized as PPDM. IPDM patients had HbA1c levels neither uniformly <8.0% nor uniformly >8.5% during FY 2012 despite receiving diabetes care.

### Baseline measures

We used FY 2012 data to characterize our cohort’s baseline demographics, comorbidities, medication use, and disease control. Demographic factors included: 1) age (continuous and categorized as <40, 40–49, 50–59, 60–69, 70–79, or ≥80 years); 2) sex (male or female); 3) race (White, Black or African-American, Asian, American Indian/Alaska Native, Native Hawaiian/Pacific Islander, or unknown); 4) ethnicity (Hispanic or Latino/a, non-Hispanic or Latino/a, or unknown); 5) marital status (currently married, never married, previously married, or unknown); 6) homelessness (yes or no); 7) copay status (no copay due disability or low income, must pay copay, or unknown); 8) distance in miles from home ZIP code to assigned VHA clinic (continuous and categorized as 0–9, 10–19, 20–29, 30–39, 40–49, 50–59, or ≥60 miles); 9) home ZIP code rurality (rural or urban); and 10) region of the country in which the assigned VHA facility was located (West, Midwest, Northeast, or South).

We utilized diagnostic cost group (DCG) scores to reflect general comorbidity. We identified specific comorbidities by the presence of relevant ICD-9 codes in VHA outpatient or inpatient files from FY 2012 (S1 Appendix). Medical comorbidities included hypertension, hyperlipidemia, coronary artery disease, congestive heart failure, cerebrovascular disease, as well as diabetic retinopathy, neuropathy, and nephropathy. Mental health comorbidities included depression, post-traumatic stress disorder, schizophrenic disorders, bipolar affective disorders, tobacco abuse, alcohol abuse, and illicit substance abuse. We used the last available FY 2012 weight and height data to examine BMI (continuous and categorized as <18, 18–24, 25–29, 30–34, 35–39, or ≥40 kg/m^2^).

Non-insulin diabetes medication classes included biguanides, sulfonylureas, thiazolidinediones, meglitinides, dipeptidyl peptidase-4 (DPP-4) inhibitors, glucagon-like peptide-1 (GLP-1) receptor agonists, and alpha-glucosidase inhibitors. We categorized insulin use as: 1) no insulin; 2) basal insulin only (neutral protamine Hagedorn (NPH) insulin, insulin glargine, or insulin detemir, with no prandial insulin prescription); 3) pre-mixed basal-prandial insulin (pre-mixed 70/30, 75/25, or 50/50 formulations); and 4) regimens including prandial insulin (regular insulin, insulin aspart, insulin lispro, or insulin glulisine). We also examined patients’ overall number of diabetes medication classes (continuous and ordinal), number of antihypertensive medication classes (continuous and ordinal), use of angiotensin converting enzyme-inhibitor (ACE-I) or angiotensin receptor blocker (ARB) medications, and use of statin medications.

Finally, we described disease control parameters for our cohort during FY 2012, specifically evaluating mean HbA1c, mean systolic and diastolic blood pressures (BP), mean cholesterol levels (total cholesterol, low-density lipoprotein (LDL) cholesterol, and high-density lipoprotein (HDL) cholesterol), and mean serum creatinine levels.

### Care utilization measures

After categorizing patients as PPDM, IPDM, or WCDM, we characterized patterns of healthcare utilization during FY 2013. Using VHA clinic stop codes (S2 Appendix) from FY 2013, we discerned clinic-based primary care, telephone-based primary care, clinic-based endocrinology (which includes any specialty diabetes care), emergency department/urgent care, home telehealth care, and clinic-based mental health care utilization. We also determined numbers of VHA inpatient hospital stays. For each utilization outcome, we limited to one utilization episode per day to avoid double counting.

### Statistical analysis

First, we conducted a cross-sectional, descriptive examination of baseline measures (demographics, comorbidities, medication use, and disease control) by diabetes control category. The descriptive cohort consisted of 435,820 patients (S3 Appendix). We then constructed a multivariable logistic regression model examining the association between patient factors and having PPDM in FY 2012, with WCDM as the reference group. We chose WCDM as the reference group for these analyses (rather than IPDM) because we were specifically interested in comparing patients with PPDM to those who responded well to clinic-based care. All covariates were selected *a priori* and were simultaneously entered into the model after assessing potential collinearity; empirical sandwich standard errors were utilized to account for clustering within home VHA facility.

We then individually examined the association between having PPDM (vs. WCDM) and each FY 2013 VHA utilization outcome, adjusting each model for baseline demographics. The analytic cohort consisted of 284,601 patients with PPDM or WCDM (S3 Appendix). All covariates were selected *a priori* and were simultaneously entered into the model after assessing potential collinearity. We fit a zero-truncated negative binomial model for clinic-based primary care, as all patients had at least one clinic-based primary care visit, and a negative binomial model for telephone-based primary care, where utilization was also common [19]. Because most of the sample did not incur utilization for clinic-based endocrinology, emergency department/urgent care, clinic-based mental health care, and home telehealth care, we fit marginal zero-inflated negative binomial models for these outcomes [20]. Due to low inpatient utilization (12.1% of the cohort with at least one admission), number of VHA hospital admissions was dichotomized as incurring an admission in FY 2013 or not; this binary outcome was modeled using logistic regression.

Associations were considered statistically significant if the p-value for the association was <0.05. Due to our large sample size, we recognized that our analysis might identify certain associations with PPDM that were statistically significant, but of insufficient magnitude to be clinically meaningful. Therefore, we chose a priori odds ratio (OR) thresholds at which we would consider associations clinically significant as well as statistically significant. Estimated OR and rate ratios (RR) were considered clinically significant if point estimates were ≤0.80 or ≥1.25.

## Results

Our descriptive cohort comprised 435,820 Veterans with type 2 diabetes who had established primary care within the VHA, and at least two HbA1c measurements during FY 2012. Approximately twelve percent of patients met PPDM criteria (n = 50,739).

### Descriptive data

Table 1 summarizes descriptive data for the entire cohort and for each diabetes control category. Patients with PPDM were younger on average than other patients with mean age 61.2 years (SD 9.7). Although our cohort was predominantly male (96.9%), the PPDM group had the highest proportion of females (3.9%). A higher proportion of patients in the PPDM group identified as Black/African-American (23.4%) and as Hispanic or Latino/Latina (7.1%) compared to other diabetes control groups. A lower proportion of patients with PPDM were married during the study period (46.3%). Homelessness was uncommon overall (2.1%), but the highest proportion among diabetes control groups occurred in PPDM (3.2%).

**Table 1:** Descriptive data for all patients with type 2 diabetes from FY 2012, also organized by diabetes control category (WCDM, IPDM, and PPDM).

Variables	All Patients (n = 435,820)	Diabetes Control Category		
		WCDM (n = 233,862)	IPDM (n = 151,219)	PPDM (n = 50,739)
**Demographics**				
Age in years, mean (SD)	64.8 (9.6)	66.2 (9.4)	63.9 (9.4)	61.2 (9.7)
Age in years (%)	0.8	0.5	0.9	1.7
<40				
40–50	4.8	3.4	5.5	9.0
50–60	17.8	14.6	19.8	26.9
60–70	50.2	50.6	50.8	46.6
70–80	18.2	20.8	16.4	11.5
≥80	7.9	9.7	6.3	4.0
Male (%)	96.9	96.9	97.1	96.1
Race (%)	71.8	73.6	71.2	65.2
White				
Black or African-American	17.5	15.8	18.2	23.4
Asian	0.5	0.5	0.5	0.6
American Indian/Alaska Native	0.7	0.6	0.7	1.0
Hawaiian/Pacific Islander	1.1	1.0	1.1	1.3
Unknown	8.1	8.1	8.0	8.2
Ethnicity (%)	5.2	4.4	5.6	7.1
Hispanic or Latino/a				
Not Hispanic or Latino/a	90.5	91.0	90.2	88.9
Unknown	4.2	4.4	4.1	3.9
Marital status (%)	59.3	61.2	58.4	53.3
Currently married				
Never married	10.9	9.9	11.3	14.4
Previously married	29.3	28.4	29.8	31.9
Unknown	0.2	0.2	0.2	0.2
Homeless (%)	2.1	1.6	2.3	3.2
Copay status (%)	55.6	56.1	55.4	54.1
No copay due to disability				
No copay due to low income	26.7	25.5	27.4	29.7
Must pay copay	16.6	17.4	16.1	14.9
Unknown copay status	0.9	0.8	0.9	1.1
Miles to assigned VHA clinic, mean (SD)	22.1 (49.8)	22.2 (49.7)	22.2 (50.2)	21.8 (48.8)
Home ZIP code in rural area (%)	17.0	17.3	16.8	15.8
Region of residence (%)	13.3	13.7	13.1	11.8
Northeast				
South	45.0	44.4	44.9	47.7
Midwest	22.9	23.1	23.0	21.2
West	18.7	18.5	18.8	19.0
**Comorbidities**				
Mean Diagnostic Cost Group score (DCG), mean (SD)	1.0 (1.3)	1.0 (1.3)	1.1 (1.4)	0.9 (1.2)
Hypertension (%)	86.0	85.7	86.6	85.3
Hyperlipidemia (%)	81.7	81.4	82.1	81.6
Coronary artery disease (%)	29.3	29.2	30.1	27.8
Congestive heart failure (%)	9.5	8.8	10.6	9.7
Cerebrovascular disease (%)	8.1	8.3	8.2	7.4
Diabetic complications (%)				
Retinopathy	15.4	10.9	19.1	25.6
Neuropathy	18.4	16.1	20.4	23.1
Nephropathy	5.0	3.9	6.1	6.5
Depression (%)	23.5	22.3	24.3	26.7
PTSD (%)	15.4	15.7	15.2	15.0
Schizophrenic disorders (%)	2.1	2.2	2.0	2.2
Bipolar disorders (%)	2.1	2.0	2.2	2.5
Tobacco abuse (%)	15.9	15.3	16.2	17.5
Alcohol abuse (%)	4.5	4.5	4.5	4.7
Substance abuse (%)	3.5	3.1	3.8	4.6
BMI in kg/m^2^, mean (SD)	32.7 (6.4)	32.2 (6.3)	33.2 (6.4)	33.4 (6.6)
BMI in kg/m^2^ (%)	0.1	0.1	0.1	0.1
<18				
18–24	8.3	9.4	6.8	7.4
25–29	28.4	30.7	26.2	24.9
30–34	31.9	31.4	32.6	31.8
35–39	18.6	17.2	20.3	20.6
≥40	12.4	10.9	13.8	14.9
**Medication use**				
Biguanide (%)	68.0	70.4	66.2	62.3
Sulfonylurea (%)	49.4	45.1	56.5	48.3
Thiazolidinedione (%)	5.3	4.9	6.3	4.3
Meglitinide (%)	0.2	0.1	0.2	0.2
DPP-4 inhibitor (%)	1.4	1.0	2.1	1.4
GLP-1 receptor agonist (%)	0.3	0.1	0.4	0.6
α-Glucosidase inhibitor (%)	2.2	1.4	3.1	3.4
Insulin use category (%)	55.4	72.7	40.1	21.4
No insulin				
Basal insulin	15.8	10.0	22.0	24.1
Pre-mixed insulin	5.7	4.0	7.0	9.2
Prandial insulin	22.9	13.0	30.7	45.0
Diabetes medication classes, mean (SD)	1.7 (0.7)	1.5 (0.6)	2.0 (0.7)	2.0 (0.8)
BP medication classes, mean (SD)	2.4 (1.4)	2.4 (1.4)	2.5 (1.4)	2.4 (1.4)
ACE-I or ARB (%)	77.9	76.8	79.1	79.5
Statin (%)	80.8	80.7	81.0	80.5

**Abbreviations**: WCDM = well-controlled diabetes mellitus; IPDM = intermittent poorly-controlled diabetes mellitus; PPDM = persistent poorly-controlled diabetes mellitus; SD = standard deviation; PTSD = post-traumatic stress disorder; BMI = body mass index; DPP-4 = dipeptidyl peptidase-4; GLP-1 = glucagon-like peptide-1; ACE-I = angiotensin converting enzyme inhibitor; ARB = angiotensin receptor blocker

A higher prevalence of diabetes complications was observed in PPDM than other diabetes control categories; 25.6% had retinopathy, 23.1% had neuropathy, and 6.5% had nephropathy. Among mental health comorbidities, the PPDM subgroup had higher rates of depression (26.7%) and tobacco abuse (17.5%) than did their better-controlled counterparts.

Patients with PPDM used more diabetes medications than other subgroups (mean 2.0 medication classes) however the specific agents varied by diabetes control category. Patients with PPDM were less likely to use metformin than other patients (62.3%). The PPDM subgroup utilized more insulin than other groups, including basal- (24.1%), premixed- (9.2%), and prandial-containing (45.0%) insulin regimens.

Table 2 presents descriptive disease control data for the entire cohort and for each diabetes control category. Expectedly, patients with PPDM had the highest mean HbA1c (10.4%).

**Table 2:** Descriptive disease control data for all patients with type 2 diabetes from FY 2012, also organized by diabetes control category (WCDM, IPDM, and PPDM).

Variables (Units)*	All Patients (n = 435,820)		Diabetes Control Category					
			WCDM (n = 233,862)		IPDM (n = 151,219)		PPDM (n = 50,739)	
	Mean	(SD)	Mean	(SD)	Mean	(SD)	Mean	(SD)
HbA1c (%)	7.8	(1.6)	6.7	(0.6)	8.3	(1.2)	10.4	(1.4)
Systolic BP (mm Hg)	134.3	(18.6)	133.56	(18.4)	134.7	(18.6)	136.0	(19.1)
Diastolic BP (mm Hg)	74.9	(11.7)	74.2	(11.6)	75.1	(11.8)	76.9	(11.9)
Total cholesterol (mg/dL)	161.3	(43.0)	156.9	(38.7)	161.9	(44.1)	173.6	(51.1)
LDL cholesterol (mg/dL)	87.6	(33.1)	85.6	(31.3)	88.1	(33.7)	95.7	(37.6)
HDL cholesterol (mg/dL)	39.7	(11.1)	40.6	(11.4)	38.6	(10.7)	39.1	(11.0)
Serum creatinine (mg/dL)	1.3	(1.0)	1.3	(1.0)	1.3	(1.0)	1.2	(0.8)

**Abbreviations**: WCDM = well-controlled diabetes mellitus; IPDM = intermittent poorly-controlled diabetes mellitus; PPDM = persistent poorly-controlled diabetes mellitus; SD = standard deviation; BP = blood pressure; mm Hg = millimeters mercury; LDL = low-density lipoprotein; mg/dL = milligrams per deciliter; HDL = high-density lipoprotein

*Missingness: systolic and diastolic blood pressure n = 23; total cholesterol n = 13,679; LDL cholesterol n = 7,646; HDL cholesterol n = 7,339; serum creatinine n = 25,153

### Clinical factors associated with PPDM

To evaluate the association of PPDM with demographic factors, comorbidities, medication use, and care utilization while accounting for underlying population differences, we used multivariable logistic regression, with WCDM patients as the reference group. The analytic cohort for multivariable modeling included 284,601 Veterans with WCDM or PPDM; these models did not include patients with IPDM. Numerous demographic factors had clinically (OR/RR point estimate ≤0.80 or ≥1.25) and statistically (α = 0.05) significant associations with PPDM (Table 3). We observed an inverse relationship between age and PPDM; using age 60–69 years as a reference, the youngest age category (<40 years) had an OR estimate for PPDM of 3.79 (95% CI: 3.45, 4.17) while the oldest age category (≥80 years) had an estimated OR of 0.57 (95% CI: 0.55, 0.61). African-American race (OR 1.45, 95% CI: 1.40, 1.50) and Hispanic or Latino/Latina ethnicity (OR 1.42, 95% CI: 1.34, 1.50) also had strong clinically and statistically significant associations with PPDM.

**Table 3:** Select model covariates showing association between patient factors and PPDM status (model includes PPDM and WCDM patients only).

Variable	OR for PPDM (95% CI)
**Demographics**	
Age (reference: 60–70)	
<40	3.79 (3.45, 4.17)*
40–50	2.96 (2.82, 3.10)*
50–60	1.86 (1.81, 1.92)*
70–80	0.61 (0.59, 0.63)*
≥80	0.57 (0.55, 0.61)*
Male sex	1.15 (1.09, 1.22)
Black or African-American race (reference: White)	1.45 (1.40, 1.50)*
Hispanic or Latino/a ethnicity (reference: non)	1.42 (1.34, 1.50)*
Never married (reference: currently married)	1.24 (1.20, 1.28)
Homeless	1.22 (1.14, 1.31)
**Comorbidities**	
BMI (reference: 18–24)	
25–29	1.00 (0.96, 1.05)
30–34	1.08 (1.04, 1.13)
35–39	1.11 (1.06, 1.17)
Hypertension	0.96 (0.93, 0.99)
Hyperlipidemia	1.05 (1.02, 1.09)
Coronary artery disease	1.07 (1.05, 1.10)
Congestive heart failure	1.07 (1.03, 1.11)
Diabetes complications	
Diabetic retinopathy	1.68 (1.63, 1.73)*
Diabetic neuropathy	1.17 (1.14, 1.21)
Diabetic nephropathy	1.26 (1.19, 1.34)*
Depression	1.07 (1.04, 1.10)
Schizophrenic disorders	0.78 (0.72, 0.84)*
Tobacco abuse	1.10 (1.07, 1.14)
Alcohol abuse	0.91 (0.86, 0.96)
**Medication use**	
Biguanide	1.18 (1.14, 1.21)
Sulfonylurea	2.58 (2.50, 2.66)*
Insulin Use Category (reference: no insulin)	
Basal insulin	8.24 (7.93, 8.57)*
Pre-mixed insulin	10.80 (10.11, 11.54)*
Prandial insulin	18.74 (17.73, 19.81)*

*Clinically significant based on OR estimate ≤0.8 or ≥1.25.

Model covariates without statistically significant associations were not presented in the Table: these included copay status, distance to assigned VHA clinic, ZIP code rurality, region of residence, diagnostic cost group score, cerebrovascular disease, PTSD, bipolar disorder, thiazolidinedione, meglitinide, dipeptidyl peptidase4 inhibitor, glucagon-like peptide 1 receptor agonist, alpha-glucosidase inhibitor, number of blood pressure medication classes, angiotensin converting enzyme inhibitor, angiotensin receptor blocker, and statin use. **Abbreviations**: BMI = body mass index; CI = confidence interval, PPDM = persistent poorly-controlled diabetes mellitus; WCDM = well-controlled diabetes mellitus

Clinically significant associations were seen for diabetic retinopathy (OR 1.68, 95% CI: 1.63, 1.73) and nephropathy (OR 1.26, 95% CI: 1.19, 1.34). Depression and tobacco abuse, while more prevalent in the PPDM subgroup, had clinically weak associations with PPDM after adjustment for other factors.

Most medication classes had both clinically and statistically significant associations with PPDM. Insulin use was strongly associated with PPDM, with a direct relationship between regimen complexity and estimated odds ratios. Insulin regimens incorporating prandial insulin had the strongest association with PPDM (OR 18.74, 95% CI: 17.73, 19.81), followed by regimens including pre-mixed insulins (OR 10.80, 95% CI: 10.11, 11.54), and basal-only regimens (OR 8.24, 95% CI: 7.93, 8.57). There was a statistically, but not clinically, significant association between the use of ACE-I/ARB medications and PPDM status (OR 1.11, 95% CI: 1.07, 1.14), and a non-significant association between statin use and PPDM (OR 1.00, 95% CI: 0.97, 1.03).

### Care utilization in association with PPDM

As seen in Table 4, utilization of primary care telephone visits, home telehealth program and endocrinology care had clinically and statistically significant associations with PPDM. While patients with PPDM more often received endocrinology care than those with WCDM, 73.6% of the PPDM population did not see endocrinology during the observation period. High utilization of the ED was likewise associated with PPDM, bordering on clinical significance. In contrast, mental health encounters were negatively associated with PPDM. No clinically significant association was observed between PPDM and primary care clinic visits or the occurrence of ≥1 inpatient stay.

**Table 4:** Healthcare utilization associated with PPDM status.

FY 2013 Utilization	Rate ratio associated with PPDM (95% CI)
Primary Care office visits	1.12 (1.11, 1.13)
Primary Care telephone visits	1.29 (1.27, 1.31)
Endocrinology/Diabetes visits	3.56 (3.47, 3.66)
Care Coordination/Home Telehealth	1.82 (1.79, 1.86)
Mental Health visits	0.82 (0.80, 0.84)
ED/UC visits	1.25 (1.22, 1.27)
≥1 Inpatient stay*	1.11 (1.10, 1.13)*

*Estimated OR associated with having at least one inpatient stay (not rate ratio).

**Abbreviations**: FY = fiscal year; PPDM = persistent poorly-controlled diabetes mellitus; ED = emergency department; UC = urgent care

## Discussion

To reduce diabetes complications and the high costs of diabetes care, patients with PPDM require alternatives to continued, insufficiently effective clinic-based management. Understanding demographics, comorbidities, and care utilization in PPDM could inform more effective strategies for this group. This analysis examined the scope of PPDM within a large, nationwide type 2 diabetes cohort. We found that nearly 12% of VHA patients with type 2 diabetes met criteria for PPDM despite engagement with diabetes care. Compared to well-controlled patients, individuals with PPDM were generally younger, more often from racial and ethnic minorities, and more often unmarried or homeless. Patients with PPDM did not have substantially more comorbid medical or mental health diagnoses compared to other patients, except for diabetes complications. Individuals with PPDM used more diabetes medications than well-controlled patients, and their treatment regimens were more complex, with greater use of prandial insulin. Those with PPDM had higher healthcare utilization, especially endocrinology care and home telehealth services.

With regard to clinical factors associated with PPDM, this cross-sectional study cannot establish causation or directionality of associations. However, our findings provide insight into care patterns for patients with persistently poor diabetes control, and encourage consideration of how current practices might evolve to more effectively serve the PPDM population.

### Findings in context of current knowledge

While poor access to care and insufficient prescription coverage may underlie many cases of suboptimal diabetes control, patients with PPDM maintain poor control despite receiving care. Because VHA provides high access to care and prescription coverage, it represents an ideal venue to examine the problem of PPDM.

PPDM patients were younger than those with better diabetes control. Other studies likewise suggest that poor diabetes control disproportionately affects younger patients [21–26]. This finding has been attributed to poorer diabetes self-care practices among younger patients, including less glucose monitoring and higher rates of dietary and medication non-adherence [22]. Interference from work and other social commitments may also contribute to worse glycemic control in younger patients. In addition to age, we demonstrated racial/ethnic disparities in diabetes control among patients with good access to care in the VHA system. African-American patients more often had PPDM (OR 1.45, 95% CI: 1.40, 1.50) compared to White patients. Similarly, ethnically Hispanic or Latino/a patients more often had PPDM (OR 1.42, 95% CI: 1.34, 1.50) than non-Hispanic or Latino/a patients. These findings are consistent with prior studies showing higher HbA1c among African-American and Hispanic or Latino/Latina patients [23, 27–30], even in individuals without diagnosed diabetes [31, 32].

Except for diabetic retinopathy and nephropathy, our data do not suggest a clinically significant association between PPDM and the presence of comorbid illnesses. The lack of such an association between depression and poor glycemic control is noteworthy, as multiple reports have linked depression with poorer glycemic control [33, 34]. One possible explanation for the lack of association between depression and PPDM in our study is that depression is more common in Veteran populations, regardless of diabetes control; the overall prevalence of depression in our study was nearly triple that in general populations (23.5% versus 8.1%) [35]. This high prevalence of depression, even among those with good glycemic control, may have masked any link between depression and diabetes control. A weaker link between depression and PPDM may therefore be a unique feature of the Veteran population. Of note, we could not evaluate depression severity, so future studies should assess whether worsening diabetes control in Veterans with PPDM parallels depression severity.

Our findings demonstrate a strong clinically and statistically significant association between complexity of diabetes medication regimens and poor diabetes control. Insulin users had PPDM much more often than non-insulin users, especially for more complex regimens including premixed insulin (OR 10.80, 95% CI: 10.11, 11.54) and prandial insulins (OR 18.74, 95% CI 17.73, 19.81). The relationship between complexity of diabetes regimen and PPDM status is likely bidirectional, as patients with type 2 diabetes typically require evidence of poor control before medication intensification occurs. However, since complex regimens are more common among patients with PPDM, one could also conclude that treatment with intensive insulin regimens may be insufficient in many cases to resolve PPDM. Although we could not examine medication adherence in this analysis, increased medication regimen complexity has been linked to non-adherence [36, 37], particularly among working adults [21]; it is therefore possible that poorer adherence to complex regimens could attenuate the benefit of intensive glucose-lowering therapy in some patients with PPDM.

Patients with diabetes are high healthcare utilizers, particularly younger patients with poor control [3, 38]. In 2017, diabetes accounted for $13.9 million in inpatient costs, $12.5 million in physician office visits, $5 million in home health services and $5.2 million in ED visits [3]. We observed higher healthcare utilization in patients with PPDM compared to better-controlled patients, where the strongest association was observed with endocrinology care visits (RR 3.56, 95% CI 3.47, 3.66). As with the likely bidirectional relationship between medication intensification and PPDM, poor glycemic control is often a prerequisite for referral to specialist care. However, since the PPDM population has poor control in the setting of higher utilization of endocrinology services, it is also possible that currently available, clinic-based VHA specialist care does not meet the needs of certain patients with PPDM. Mental health visits (RR 0.82, 95% CI 0.80, 0.84) were inversely associated with PPDM in our study, suggesting that care for comorbid mental illness may protect against PPDM.

### Implications of findings

A central theme that emerges from our data is that patients with PPDM are more likely to receive intensive medication regimens and specialty diabetes care, yet remain poorly controlled. While there is likely a bidirectional relationship between PPDM and use of complex insulin regimens or specialist care as above, this theme suggests that current care approaches may be insufficient to address poor glycemic control for some patients with PPDM.

By contrast, our findings simultaneously suggest that some patients with PPDM may actually be underutilizing care. For instance, only 62.3% of patients with PPDM used metformin, which remains indicated even among patients on insulin. Similarly, despite a mean HbA1c of 10.4%, only 45.0% of the PPDM population was prescribed prandial insulin. Additionally, a large majority (73.6%) of PPDM still did not visit with endocrinology during the observation period, and could potentially benefit from specialty diabetes care. While there may be valid reasons why some patients do not use available medical therapies (e.g. intolerance, contraindication, refusal, insufficient self-management skills) and specialty care (e.g. inability to travel to clinic visits, lack of available endocrinologist, refusal of subspecialty care), it is possible that greater utilization of these ‘standard’ approaches to glycemic management could benefit some patients with PPDM.

Further longitudinal research could clarify the apparent tension between inadequacy of current approaches to care in PPDM and possible underutilization of the same processes within the PPDM population. In the meantime, based on our current data, we would hypothesize that PPDM is a heterogeneous population with differing needs related to medication management and care delivery. For example, some patients with PPDM would benefit from treatment intensification, while medication escalation may be unhelpful for others. Similarly, some PPDM patients may benefit from additional support from services like home telehealth, while others do not. Further research is needed to understand factors that predict response to available care approaches in PPDM, as well as why more patients with PPDM are not using available services like endocrinology and home telehealth. Because use of newer diabetes medication classes like GLP-1 receptor agonists and sodium-glucose cotransporter-2 inhibitors was low in our population, another area for future research would be exploring the value of these agents in PPDM, as they can reduce insulin burden and potentially allow simplification of insulin regimens. Ultimately, management strategies for PPDM should consider these patients’ complex needs, as it is unlikely that all patients with PPDM benefit from the same approaches.

In addition to considering which clinical approaches benefit patients with PPDM, we must also consider how those interventions are delivered. Patients with PPDM are younger than well-controlled patients, so may be more vulnerable to time constraints imposed by work and social engagement. Given these possible limitations, PPDM may represent an ideal population for telemedicine- and web-based strategies to deliver education, self-care counseling and medication management. Such strategies can be effective in improving glycemic control in both VA and non-VA populations [39–42], including among Veterans with PPDM [39]. For patients that may benefit from specialty care, but are not seeing endocrinology due to difficulty attending regular clinic visits, telemedicine could broaden the reach of specialist care.

Overall, our findings suggest that even within the VHA, where patients can readily access care, PPDM represents a difficult challenge. A system of care that enables recognition of factors underlying PPDM and allocation of patients to individualized care approaches may more effectively manage the burden of PPDM.

### Limitations

This study had several strengths, including its large sample size, nationwide cohort, consideration of demographics, multiple medical/mental health comorbidities and assessment of medication use and care utilization. Additionally, we used clinically relevant criteria based on American Diabetes Association (ADA) guidelines to categorize diabetes control and account for patient’s recent diabetes care. By analyzing PPDM, we had the unique opportunity to study patient and care-related factors that influence glycemic control in a population of patients who were actively utilizing care, thus eliminating access to care as a confounder. While studying a population with good access to care is helpful when trying to isolate causes of poor glycemic control, our findings’ lack of generalizability to non-VA and non-primary care linked populations is a limitation. Generalizability to current practice is also a limitation, as this study examined data from FY 2012–2013. Additional limitations include low female representation, lack of data regarding dual use of VA and non-VA hospitals, inability to establish causal links due to cross-sectional design, and not accounting for additional factors that could influence diabetes control and/or indicate a need for more intensive approaches to care (e.g. depression severity, diabetes duration).

The prevalence of PPDM, as well as associated factors and care utilization patterns are not well understood outside the VA. More research is needed to fill this knowledge gap. Tailoring approaches to PPDM will also require an enhanced understanding of long-term outcomes in this group, and how utilization of certain medications and care strategies influence these outcomes. Finally, although examining associations and care utilization within the IPDM population was beyond the scope of this manuscript, the large group of patients with IPDM is an important target for future study.

## Conclusion

Patients with PPDM remain poorly controlled despite greater use of complex medication regimens and outpatient services. It is likely that PPDM is a function of both the inadequacy of current care approaches and underutilization of available care. Novel approaches to PPDM must facilitate recognition of the factors that perpetuate individual cases of PPDM, and appropriately allocate patients to treatments and care delivery strategies that meet their specific needs.

## Supporting information

S1 Appendix ICD-9 codes for comorbid conditions. (DOCX)

S2 Appendix Veterans Health Administration (VHA) stop codes for FY 2013. (DOCX)

S3 Appendix Construction of analytic cohort. (DOCX)
