# Supplementary material for: Clinical factors associated with persistently poor diabetes control in the Veterans Health Administration: A nationwide cohort study
Source: PLoS One. 2019 Mar 29;14(3):e0214679. doi: 10.1371/journal.pone.0214679 (PMC6440639; doi:10.1371/journal.pone.0214679)
Supplement: S1 Appendix — (DOCX) [file pone.0214679.s001.docx]

**S1 Appendix: ICD-9 codes for comorbid conditions**

| Diabetes  Type 2  Type 1 | 250.*0, 250.*2  250.*1, 250.*3 |
| --- | --- |
|  |  |
| Hypertension | 401.0, 401.1, 401.9, 405.01, 405.09, 405.11, 405.19, 405.91, 405.99 |
| Hyperlipidemia | 272.0, 272.2, 272.4 |
| Tobacco abuse | 305.1 |
| Obesity* | 278.00, 278.01 |
| Overweight* | 278.02 |
|  |  |
| Microvascular complications:  Retinopathy  Neuropathy  Nephropathy | 362.01--.06, 250.50, 250.51, 250.52, 250.53  357.2, 250.60, 250.61, 250.62, 250.63  250.40, 250.41, 250.42, 250.43 |
|  |  |
| Coronary artery disease | 414.00--.07, 414.2--.9, 410.*, 412.* |
| Congestive heart failure | 402.01, 402.11, 402.91, 404.*, 425.11--.9, 428.* |
| Peripheral vascular disease | 440.*, 443.89, 443.9, 557.1, 557.9 |
| Cerebrovascular disease | 433.*, 434.*, 435.8, 435.9, 436.*, 437.0, 437.8, 437.9, 438.13, 438.14, 438.81, 438.82, 438.89, 438.9 |
|  |  |
| Depression | 296.2--.26, 296.3-.36, 296.89, 300.4, 309.0, 309.1, 311.* |
| PTSD | 309.81 |
| Schizophrenic disorders | 295.*, V11.0 |
| Bipolar disorders | 296.00--.06, 296.40--.8, 296.89 |
| Eating disorders | 307.1, 307.50, 307.51, 307.59 |
| Alcohol abuse | 291.*, 303.90, 303.91, 303.92, 305.00, 305.01, 305.02 |
| Other substance abuse | 304.*, 305.20--.92 |

*Obesity and overweight are primarily based on body mass index (BMI): overweight 25-29.9 kg/m^2^, obesity ≥ 30 kg/m^2^.
