# Supplementary material for: Clinical factors associated with persistently poor diabetes control in the Veterans Health Administration: A nationwide cohort study
Source: PLoS One. 2019 Mar 29;14(3):e0214679. doi: 10.1371/journal.pone.0214679 (PMC6440639; doi:10.1371/journal.pone.0214679)
Supplement: S2 Appendix — (DOCX) [file pone.0214679.s002.docx]

**S2 Appendix:** **Veterans Health Administration (VHA) stop codes used for FY 2013**

| **Outcome** | **VA data source & definition** | **Fee basis definition** |
| --- | --- | --- |
| ED visits count. *Limit to 1 per day.* | Any records from MedSAS outpatient (a) with credit stop codes 130, 131 OR (b) with primary stop codes 130, 131 but none of the following credit stop codes: 107, 115, 152, 311, 321, 328, 329, 333, 334, 430, 435, 474, 999 | FPOV = 32, 33; PLSER = 23; CPT1 = 99281, 99282, 99283, 99284, 99285, 99288  ANCIL: FPOV, CPT1, PLSER  INPT: FPOV, CPT1  MED: FPOV, CPT1, PLSER |
| Office-based Primary Care visits count. *Limit to 1 per day.* | 322, 323, 342, 348 stop codes from MedSAS outpatient | VA only |
| Telephone-based Primary Care visits count. *Limit to 1 per day.* | 338 stop code from MedSAS outpatient | VA only |
| Mental health visits. *Limit to 1 per day.* | 500-599 stop codes from MedSAS outpatient | VA only |
| Hospitalizations – Binary, yes/no whether an admission occurred. *Exclude nursing home/long term care* | MedSAS inpatient | One or more records in Fee basis inpatient (INPT) file (do not look in ANCIL) |
| Specialty care visits not including endocrinology. *Limit to 1 per day.* | 300-399 stop codes, excluding those mentioned elsewhere (i.e., excluding 305, 306, 322, 323, 338, 342, 348), from MedSAS outpatient | VA only |
| Endocrinology visits and specialty diabetes care. *Limit to 1 per day.* | 305, 306 stop codes from MedSAS outpatient | VA only |
