# Supplementary material for: Clinical factors associated with persistently poor diabetes control in the Veterans Health Administration: A nationwide cohort study
Source: PLoS One. 2019 Mar 29;14(3):e0214679. doi: 10.1371/journal.pone.0214679 (PMC6440639; doi:10.1371/journal.pone.0214679)
Supplement: S3 Appendix — (DOCX) [file pone.0214679.s003.docx]

**S3 Appendix:** Construction of analytic cohort.

Unique patients with ≥2 HbA1c measurements in FY 2012 and ≥1 in FY 2013

N = 488,786

PPDM

N=50,739

Unique patients with ≥2 outpatient or ≥1 inpatient visit* with 250.xx ICD-9 code in FY 2012

N = 1,049,638

WCDM

N=233,862

IPDM

N=151,219

Patients not in Puerto Rico in FY 2012

N = 467,779

Patients not assigned to a station more than 1000 miles from their home

N= 477,236

Patients assigned to a station in the 50 United States, Washington, DC, or Puerto Rico

N= 479,693

Patients with unique CDW identifiers

N=703,887

Patients with non-missing BMI between of 13 and 85 kg/m^2^

N= 482,940

N= 488,752

Patients with ≥1 diabetes-related ICD-9 code (250.xx) in FY 2012

N= 488,752

Unique patients with age ≥18 by start of FY 2012

N = 723,748

Unique patients in FY 2012 with VHA outpatient and/or inpatient encounters

N = 5,774,903

Unique patients with ≥1 pharmacy fill from VA drug classes HS501 or HS502 in FY 2012

N = 830,602

Unique patients with ≥1 primary care visit in FY 2012 and FY 2013 (stop codes in S2 Appendix)

N = 738,317

Unique patients assigned to a station with 100 or more patients in FY 2012

N = 719,370

Unique patients with ≥1 outpatient primary care encounter in FY 2012 with MD, PA, NP or MD-RES provider code

N = 710,267

Unique patients with ≥1 outpatient visit in FY 2013 with 250.xx ICD-9 code

N = 723,752

**Descriptive cohort.** Patients with 1) any FY 2012 comorbidity and medication data; 2) age ≥18 at start of FY 2012; 3) assignment to US-based station, and 4) type 2 diabetes

N= 435,820

**Analytic cohort**

N = 284,601

*Any duplicate visits for the same clinic on the same day were removed. A count of the total number of unique days a subject went to a clinic was created. **Abbreviations:** FY=fiscal year; VHA=Veterans Health Administration; ICD-9=International Classification of Diseases, 9^th^ revision; MD=medical doctor; PA=physician assistant; NP=nurse practitioner; MD-RES=resident medical doctor; CDW=corporate data warehouse; HbA1c=hemoglobin A1c; BMI=body mass index; DC=District of Columbia; US=United States; WCDM=well-controlled diabetes mellitus; IPDM=intermittent poorly-controlled diabetes mellitus; PPDM=persistent poorly-controlled diabetes mellitus
